# Extracorporeal hemoadsorption therapy as a potential therapeutic option for rapid removal of Apixaban in high risk-surgical patients: a case report

**DOI:** 10.1186/s13256-023-03949-3

**Published:** 2023-07-07

**Authors:** Vittorio Dalmastri, Andrea Angelini, Vera Minerva, Melissa Ballarini, Francesco Grammatico, Paola Todeschini, Attilia Maria Pizzini, Mauro Silingardi, Gaetano La Manna

**Affiliations:** 1 Nephrology Dialysis and Renal Transplant Unit IRCCS St, Orsola University Hospital, Bologna, Italy; 2 Department of Medicine, Maggiore General Hospital, AUSL, Bologna, Italy; 3 Dialysis Unit, Maggiore General Hospital, National Public Health System, Largo Nigrisoli, 2, 40133 Bologna, BO Italy

**Keywords:** Apixaban, Hemoadsorption, CytoSorb

## Abstract

**Background:**

Apixaban is a non-vitamin K antagonist oral anticoagulant (NOACs) recently emerged as an effective alternative to conventional vitamin K antagonists (VKAs) in the treatment of several thromboembolic disorders. However, in case of overdose or in patients requiring emergency surgery there is a high bleeding rate and severe adverse side effects due to the absence of an antidote. There is promising data from *in vitro* and clinical studies, that certain antithrombotic agents (that is Rivaroxaban and Ticagrelor) have been successfully removed by the extracorporeal hemoadsorption therapy CytoSorb. Here, we present the case of a patient successfully treated with CytoSorb as a kind of antidote to enable emergency surgery for bilateral nephrostomy.

**Case presentation:**

A 82-year-old Caucasian man was admitted to the Emergency Room with acute kidney injury (AKI) in the context of severe bilateral hydroureteronephrosis. The patient’s medical history included chronic obstructive pulmonary disease, arterial hypertension, atrial fibrillation (anticoagulated with Apixaban) and a locally advanced prostate adenocarcinoma treated with trans-ureteral resection of the bladder and radiotherapy in the previous months. The indication for a bilateral nephrostomy could not be considered immediately given the major bleeding risk due to Apixaban, which was discontinued and replaced with calciparin. After 36 hours of continuous renal replacement therapy (CRRT), the Apixaban blood level was still elevated and it was decided to install CytoSorb into the running CRRT to accelerate the drug clearance. After 2 hours 30 minutes, there was good reduction of Apixaban from 139 to 72 ng/ml (reduction rate of 48.2%) registered, and this allowed for an easy placement of bilateral nephrostomies without complications. Four days after surgery renal function parameters further normalized, the patient did not require additional dialysis treatments and Apixaban therapy was prescribed again once the patient returned home.

**Conclusions:**

In this case we report the findings of a patient with post-renal AKI requiring emergency nephrostomy placement while on chronic anticoagulation with Apixaban therapy. Combined treatment with CRRT and CytoSorb was associated with the rapid and effective removal of Apixaban allowing for prompt and urgent surgery while simultaneously ensuring the low risk of bleeding as well as an uneventful post-operative course.

## Introduction

Due to its advantageous mode of action, safety profile, limited clinically relevant interactions as well as predictable pharmacokinetic and pharmacodynamic effects enabling the administration of fixed doses and the lack of frequent monitoring requirements [1], the DOAC (Direct Oral Anticoagulant) Apixaban has emerged as an effective alternative to conventional vitamin K antagonists (VKAs) in the treatment of several thromboembolic disorders and is now widely used across various indications, including the prevention of stroke and systemic embolism in patients with non-valvular atrial fibrillation (AF) and the treatment of venous thromboembolism (VTE) [2]. Following administration, the drug is rapidly absorbed, with maximum concentrations occurring 3–4 hours after oral administration and a half-life of approximately 12 hours. There are multiple elimination pathways including metabolism, biliary excretion, direct intestinal excretion, and renal excretion [3]. Drug half-life of 12 hours, however, is only achieved when the glomerular filtration rate (GFR) is > 80 ml/min/1.73 m^2^, and may be significantly longer in acute or end-stage renal failure due to renal excretion of approximately 27%. On the other hand, there is no fast and effective reversal agent available. In case of overdose or in patients requiring emergency surgery who are on regular Apixaban, this also means that there is a higher bleeding rate and more adverse side effects which are associated with increased morbidity and mortality. Due to its high degree of protein binding of 87–93% (mainly albumin), hemodialysis only has a limited impact on Apixaban exposure (approximately 14%) and therefore conventional dialysis based approaches are not recommended as an effective method of managing Apixaban overdose [4]. Current guidelines require several days of Apixaban discontinuation prior to surgical intervention, which is, however, not feasible in emergency situations. The antidote (Andexanet alfa) is commercially available even though there is no official indication for preventive use in patients requiring emergency surgery. There is promising data from in vitro but also clinical studies, that certain DOACs (that is Rivaroxaban) and the antiplatelet Ticagrelor have been successfully removed by a new hemoadsorption device (CytoSorb, CytoSorbents Inc.) [5–7]. Due to its structural similarities, the assumption is obvious that Apixaban might also be eliminated in a comparable manner. In fact, CytoSorb has been proven to be effective in removing Rivaroxaban during emergency cardiac surgery [6] and has been used successfully to remove Apixaban in a similar case [8]. The efficacy of CytoSorb on Apixaban has also been demonstrated in an *in vitro* study [9]. Here, we present the case of a patient in whom CytoSorb therapy was successfully applied as a kind of antidote to enable emergency surgery in a patient with acute renal failure requiring bilateral nephrostomy.

## Case report/case presentation

**Fig. 1:**
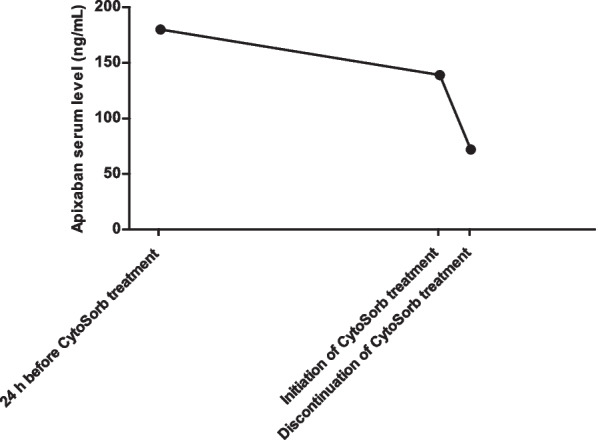
Apixaban serum level under CytoSorb hemoadsorption therapy. In the first part, drug clearance is slowed by renal failure; with the use of CytoSorb a rapid Apixaban removal was achieved with a reduction rate of 48% in two and a half hours of treatment

An 82-year-old Caucasian patient was admitted to the Emergency Room of Maggiore Hospital Bologna, Italy for post-renal AKI failure and nausea. Previously, he had suffered several days of oliguria. In addition, the day before he had had a follow-up CAT Scan for prostate cancer, revealing a bilateral renal pelvis ectasia with II-III grade hydroureteronephrosis. On admission, oligo-anuria was confirmed despite bladder catheter placement and blood tests showed severe impairment of kidney function with a serum creatinine of 18.38 mg/dl, urea 218 mg/dl, as well as mild hyperkalemia (5.6 mmol/l) and metabolic acidosis. Further medical history included hypertension, chronic obstructive pulmonary disease, atrial fibrillation on oral anticoagulant therapy with Apixaban (5 mg/twice a day), trans-ureteral resection of the bladder (TURB) and radiotherapy for incidental prostatic neoplasia. The month before he had already undergone an endoscopic ureterotomy for a ureteral stenosis (malignant progression). At the urological consult, the decision was made for a bilateral nephrostomy, however not within the next 48 hours given the major bleeding risk due to Apixaban, which was discontinued immediately and replaced with calciparin. Nevertheless, as dialysis was indicated, a hemodialysis catheter was placed in the right femoral vein (positioning in the jugular vein was not practicable due to the high risk of bleeding) and continuous renal replacement therapy (CRRT) was initiated in continuous veno-venous hemodiafiltration mode (CVVHDF) (Prismax with ST150 set, Baxter) with a bolus of low molecular weight heparin (4000 UI) as circuit anticoagulation to bridge the patient until positioning of nephrostomies could be performed. The following day, an Apixaban blood assay (measured by liquid chromatography–mass spectrometry method, CSI 5100®) confirmed still elevated blood drug values of 180 ng/ml (peak 102–416 ng/ml; through 42–183 ng/ml) and the operation was postponed once more. The following morning, another test was performed showing that the Apixaban dosage was still high (139 ng/ml). In the absence of an available countermeasure, a CytoSorb hemoadsorption column was installed into the running CRRT circuit to enable prompt intervention, without waiting for physiologic drug clearance. A new 8h-CVVHDF session was prescribed: blood flow rate was 150–200 ml/min and the adsorber was connected post-hemofilter (AN69ST membrane) in series in the hemodialysis circuit. No intradialytic anticoagulation was administered. Treatment with CytoSorb was interrupted after 2 hours and 30 minutes due to adsorber coagulation. However, he continued the pre-operative dialysis treatment for 5 hours and 30 minutes without CytoSorb. At the end of treatment, the Apixaban level was 72 ng/ml, which represents a reduction rate of 48.2% (Fig. 1). This level finally allowed for the easy placement of bilateral nephrostomies without complications. In fact, the hemoglobin levels remained stable during the entire peri- and post-operative period. As a result of the emergency nephrostomy placement, large quantities of urine were drained (about 1500 ml per nephrostomy per day). Plasma concentrations of creatinine and urea gradually decreased, so that the femoral catheter could be removed 4 days after surgery, while renal function parameters further normalized and returned to baseline values 7 days after the procedure (serum creatinine 0.76 mg/dl, urea 29 mg/dl). During the follow-up, the patient made good progress without the necessity for additional dialysis. Apixaban therapy was prescribed again once the patient returned home. The trend of the patient’s clinical parameters are summarized in Table 1.

**Table 1:** Biochemical parameters on admission, during CVVHD, after CytoSorb treatment and upon 7 days follow-up

Parameter	Admission	CVVHD 12 hours	CVVHD 36 hours	CVVHD + CytoSorb 2.5 hours	7 day follow-up
Serum Apixaban (ng/ml)	Not available	180	139	72	Not available
Hemoglobin (mg/dl)	10.2	10	9.3	9	9
Serum creatinine (mg/dl)	18.4	10.1	6.8	5.92	0.8
Urea (mg/dl)	218	159	87	Not available	29

## Discussion/conclusion

In the present case we report the findings of patient with acute kidney failure requiring emergency nephrostomy placement while being on chronic anticoagulative Apixaban therapy. Combined treatment with CRRT and CytoSorb hemoadsorption therapy was associated with a rapid and effective reduction of Apixaban plasma levels allowing for prompt and urgent surgery while simultaneously ensuring a low risk of bleeding as well as an uneventful post-operative period.

Apixaban is a direct FXa inhibitor that has been approved in many countries for several indications [10] and represents an important alternative to existing anticoagulant therapies with a favorable risk–benefit profile. However, in cases that require emergency surgery, there is no credible reversal agent available [11]. While a reversal agent for Rivaroxaban as well as Apixaban is approved in the US (Andexanet alfa/AndexXa), its safety and efficacy is not so well-established, and in 2015, post-marketing assessments showed liver toxicity, so that the medication is contraindicated in patients with significant liver disease and end-stage kidney disease [12]. Therefore, suitable measures to rapidly reverse the anticoagulant effect of Apixaban are highly desireable. In our case focusing on the approach for removal of the anticoagulant Apixaban by combining hemodialysis with off-label use of CytoSorb hemoadsorption, an emergency urological procedure crucial to resolve post-renal AKI was finally possible. Clotting of the adsorber, which occurred after the first 2 hours, was found to be the result of non-administration of an intradialytic anticoagulant, and the concomitant removal of Apixaban from the whole blood. Besides this, no other device-related adverse events were reported during or after the treatment.

The CytoSorb hemoadsorption device consists of polyvinylpyrrolidone-coated polystyrene–divinylbenzene copolymer beads able to nonspecifically bind mostly hydrophobic substances up to a molecular weight of 60 kDa on to the internal pore surface by hydrophobic interactions. Although initially intended for the use in hyperinflammatory states to remove elevated cytokine concentrations from blood (for example sepsis), the technology has meanwhile been shown to also eliminate other endogenous (bilirubin and myoglobin) and exogenous (toxins and certain drugs) substances yielding promising clinical outcomes in a variety of indications [13–16].

The presented results suggest that CytoSorb hemadsorption could potentially offer a valid option to rapidly reverse the anticoagulant effect of Apixaban. However, additional testing and studies are needed to verify these findings. Additionally, the removal of Apixaban through Cytosorb should be evaluated in emergency medicine or in the Intensive Care setting where surgery or required procedures may be at risk and/or slowed down for critically ill patients due to DOAC therapy.

## Data Availability

All data generated or analyzed during this study are included in this article and can be obtained from the corresponding author upon reasonable request.
